# Effects of NR1 splicing on NR1/NR3B-type excitatory glycine receptors

**DOI:** 10.1186/1471-2202-10-32

**Published:** 2009-04-06

**Authors:** Nora A Cavara, Angela Orth, Michael Hollmann

**Affiliations:** 1 Department of Biochemistry I – Receptor Biochemistry, Ruhr University Bochum, Universitätsstr. 150, D-44780 Bochum, Germany; 2 International Graduate School of Neuroscience (IGSN), Ruhr University Bochum, Bochum, Germany; 3 Ruhr-University Research School, Ruhr University Bochum, Bochum, Germany; 4 DFG Graduate School 736, Ruhr University Bochum, Bochum, Germany

## Abstract

**Background:**

N-methyl-D-aspartate receptors (NMDARs) are the most complex of ionotropic glutamate receptors (iGluRs). Subunits of this subfamily assemble into heteromers, which – depending on the subunit combination – may display very different pharmacological and electrophysiological properties. The least studied members of the NMDAR family, the NR3 subunits, have been reported to assemble with NR1 to form excitatory glycine receptors in heterologous expression systems. The heterogeneity of NMDARs *in vivo *is in part conferred to the receptors by splicing of the NR1 subunit, especially with regard to proton sensitivity.

**Results:**

Here, we have investigated whether the NR3B subunit is capable of assembly with each of the eight functional NR1 splice variants, and whether the resulting receptors share the unique functional properties described for NR1-1a/NR3. We provide evidence that functional excitatory glycine receptors formed regardless of the NR1 isoform, and their pharmacological profile matched the one reported for NR1-1a/NR3: glycine alone fully activated the receptors, which were insensitive to glutamate and block by Mg^2+^. Surprisingly, amplitudes of agonist-induced currents showed little dependency on the C-terminally spliced NR1 variants in NR1/NR3B diheteromers. Even more strikingly, NR3B conferred proton sensitivity also to receptors containing NR1b variants – possibly via disturbing the "proton shield" of NR1b splice variants.

**Conclusion:**

While functional assembly could be demonstrated for all combinations, not all of the specific interactions seen for NR1 isoforms with coexpressed NR2 subunits could be corroborated for NR1 assembly with NR3. Rather, NR3 abates trafficking effects mediated by the NR1 C terminus as well as the N-terminally mediated proton insensitivity. Thus, this study establishes that NR3B overrides important NR1 splice variant-specific receptor properties in NR1/NR3B excitatory glycine receptors.

## Background

Ionotropic glutamate receptors mediate most of the excitatory neurotransmission in the vertebrate central nervous system (CNS) [1]. Members of the complex subfamily of NMDARs (N-methyl-D-aspartate receptors) require glycine as a coagonist in addition to glutamate [2,3] and a pre-depolarisation of the membrane to release their block by Mg^2+ ^ions [3,4]. Via this coincidence detection, NMDARs are thought to provide the molecular basis for synaptic plasticity mechanisms like LTP and LTD [5,6], which in turn underlie higher cognitive functions like memory formation and learning. NMDARs assemble from combinations of NR1, NR2, and NR3 subunits. In a "conventional" NMDAR, two glycine-binding NR1 subunits and two glutamate-binding NR2 subunits form a tetrameric channel that – once activated – is highly permeable for Ca^2+^.

Recently, a novel type of "NMDA" receptor has been described that involves the still poorly understood NR3 subunits. As shown by Chatterton et al., both NR3A and NR3B assemble with NR1-1a to form receptors that are fully activated by glycine alone [7]. The NR1-1a/NR3 diheteromers are neither blocked by Mg^2+ ^nor permeable for Ca^2+ ^and desensitize rapidly if NR3A is present in the complex [7]. The NR3B subunits have been shown to also attenuate current amplitudes [8], and reduce Ca^2+ ^permeability of "conventional" NR1/NR2 receptors [9,10], but whether they exist in the form of an excitatory NR1/NR3B glycine receptor *in vivo *is still controversial.

NR1 as the compulsory subunit is expressed ubiquitously in the CNS [3]. Alternative N- and C-terminal splicing generates eight functional isoforms from the single gene transcript [11,12]. N-terminally, exon 5 can be inserted at position 173 [4], as indicated by the letter "b" (presence of exon 5) or "a" (absence of exon 5) in the name of the variant. NR1a splice variants lacking the encoded 21 amino acids are tonically inhibited by protons in the range of physiological pH values [13-15]. Splicing within exons 21 and 22 (coding the C-terminal cassettes C1 and C2, respectively) generates four different C-terminal variants: Deletion of exon 21 removes 111 base pairs (bp) in the C-terminal domain of NR1–2, but leaves the far C terminus (encoded by exon 22) identical to that of NR1-1. The use of an alternative splice acceptor site in exon 22 deletes 356 bp, including the stop codon, and transfers 66 bp of the previously untranslated 3' region to coding sequence (C2' cassette). Thus, exon 20 (in NR1–4) or exon 21 (in NR1–3) are followed by the 3'-end of exon 22 and the alternative C terminus encoded by a stretch of previously untranslated sequence [4,11,12,16-18].

Efficiency of export from the endoplasmatic reticulum (ER) differs for the C-terminal variants. The C1 cassette features an ER retention motif, impeding surface expression of NR1-1 and NR1–3 isoforms. In the case of NR1–3 variants, lower export efficiency might be compensated by the presence of a PDZ binding motif in the C2' cassette and the subsequent interaction with PDZ proteins. As neither NR1–2 nor NR1–4 contain the C1 cassette, none features the retention signal, and NR1–4 in addition has the C2'cassette PDZ interacting motif [19-21]. Evidence exists for a region-specific localization of NR1 splice variants in the rodent brain [22,23], but there is also considerable overlap in the expression of mRNA for the different isoforms. Strikingly, high expression of NR1b variants has been reported in structures associated with motor control [22], while NR3B has been shown to be highly expressed (and possibly restricted to) somatic motor neurons [8].

The functional heterogeneity of conventional NR1/NR2-containing NMDARs, which is in part conferred to the receptor via the NR1 subunit, consequentially raises the question about a potentially splice variant-specific interaction of NR1 with NR3. Although a recent study on a limited subset of NR1"a" variants suggests functional assembly with NR3A and NR3B [24], the most extensively characterized diheteromer features the NR1-1a isoform. Considering the impaired ER export of particularly the NR1-1a splice variant and the resulting poor membrane insertion, the reported glycine receptors featuring this NR1 isoform might be of limited relevance *in vivo*. We therefore asked whether NR3 is capable of assembly with each of the eight functional NR1 splice variants, and if so, whether the resulting receptors share the unique functional properties described for NR1-1a/NR3.

## Methods

### Accession numbers

The following clones were used: NR1-1a (GenBank:U08261), NR1-1b (GenBank:U08263), NR1–2a (GenBank:U08262), NR1–2b (GenBank:U08264), NR1–3a (GenBank:U08265), NR1–3b (GenBank:U08266), NR1–4a (GenBank:U08267), NR1–4b (GenBank:U08268), NR2B (GenBank:U11419), NR3B (GenBank:NM130455).

### cRNA synthesis

cRNA synthesis was performed as described previously [25]. Briefly, cRNA was synthesized from 1 μg of linearized DNA using an *in vitro *transcription kit (Fermentas) with a modified protocol employing 400 μM GpppG (GE Healthcare, Freiburg, Germany) for capping and an extended reaction time of 3 h with T7 polymerase.

### Electrophysiological measurements in *Xenopus laevis *oocytes

Oocytes of *Xenopus laevis *frogs (Nasco, Fort Atkinson, WI) were surgically removed from the ovaries and defolliculated as described previously [25]. They were maintained in Barth's solution supplemented with 100 μg/ml gentamicin, 40 μg/ml streptomycin, and 63 μg/ml penicillin. Selected oocytes of stages V-VI were injected with 6 pmol of cRNA for each receptor subunit (NR1 to NR3 ratio: 1:1; NR1 to NR2 ratio: 1:1; both cRNAs were mixed before injection in a standard volume) using a nanoliter injector (World Precision Instruments, Sarasota, FL). Four to 6 days after injection [12], oocyte current responses were recorded in normal frog Ringer's solution (NFR) (115 mM NaCl, 2.5 mM KCl, 1.8 mM CaCl_2_, and 10 mM HEPES-NaOH, pH 7.2) under voltage clamp at -70 mV holding potential with a TurboTec 10CX amplifier (npi Electronic, Tamm, Germany) controlled by Pulse software (HEKA Elektronik, Lambrecht, Germany). Recording pipettes were pulled from borosilicate glass (Hilgenberg, Malsfeld, Germany). Voltage electrodes had resistances of 0.5–1 MΩ and were filled with 3 M KCl; current electrodes had resistances of 0.5–1 MΩ and were filled with 3 M CsCl. Agonists and antagonists (300 μM glutamate, 10 μM glycine, 0.5 mM Mg^2+^, 5 μM CNQX, 100 μM kynurenic acid) were prepared in NFR and applied for 20 s by superfusion at a flow rate of 5 ml/min. Agonist concentrations were chosen to ensure maximal activation of both types of diheteromers, while keeping parameters constant for both types of receptors: For NR1/NR2 diheteromers, saturation is reached around 150 μM glutamate and 10 μM glycine [12], whereas NR3B-containing diheteromers desensitize at glycine concentrations above 10 μM [7]. Exemplary recordings for NR1–3a and NR1–3b with NR2B corroborated these values for our experimental design: application of four different glutamate concentrations (50 μM, 100 μM, 300 μM, 500 μM, each together with 10 μM glycine) did not lead to significantly different current amplitudes (n = 5/combination, data not shown). On the other hand, increasing the concentration of glycine from 10 μM to 150 μM reduced current responses of NR1/NR3B diheteromers approximately two- to threefold (for NR1–3a/NR3B: I_Gly150/Gly10 _= 0,47 ± 0,19; for NR1–3b/NR3B: I_Gly150/Gly10 _= 0,32 ± 0,05, n = 5).

**Figure 1:**
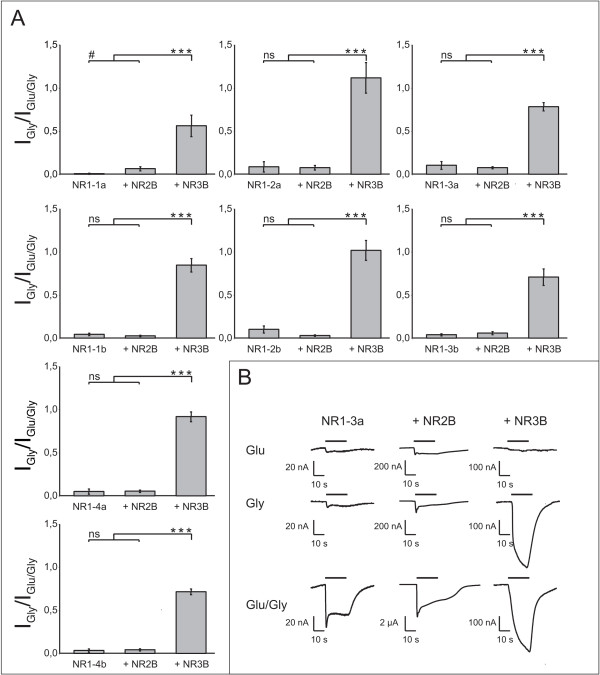
**Comparison of agonist-induced currents for all NR1 splice variant/NR3B combinations**. **A**. Ratio of current responses elicited by application of glycine (10 μM, Gly) and glycine plus glutamate (300 μM, Glu) for NR1 subunits expressed alone, NR1/NR2B heteromers, and NR1/NR3B heteromers. Data shown here are mean values ± SEM, n = 10–23 from 2–5 independent experiments per combination; *p < 0.05; **p < 0.01; ***p < 0.005 (Student's t-test). **B**. Representative current traces of diheteromers, shown exemplary for combinations with NR1–3a. Black bars denote agonist application.

Current-voltage relationships were determined between -150 mV and +50 mV and corrected for background conductivities. Data presented here are reported as mean ± SEM. Statistical significance was determined with an unpaired Student's t-test. For potentiation factors (Fig. 1A, C), error bars include the Gaussian error propagation. For normalization, amplitudes of single oocytes were divided by the mean amplitudes of an appropriate reference subunit combination, calculated separately for each agonist or antagonist. The reference combination (e.g., the NR1-1a-containing diheteromer) thereby is set to the value "1", and all other amplitudes are given relative to this.

## Results

### Excitatory glycine receptors can form with each functional NR1 splice variant

To address the question whether NR3B can interact with each of the functional NR1 splice variants to form an excitatory glycine receptor, we coexpressed each NR1 variant separately and together with either NR2B or NR3B in *Xenopus laevis *oocytes. The "conventional" NR1/NR2 heteromers in all cases gave robust current responses upon coapplication of glutamate and glycine (Fig. 1B). Significantly smaller current responses were measured when either agonist was applied alone. The same agonist profile was recorded when NR1 subunits were expressed alone (Fig. 1B). However, when NR1 was expressed together with NR3B, glycine alone was able to fully activate the receptors, regardless of the NR1 splice variant involved. By contrast, glutamate alone was not able to elicit any current responses from any NR1/NR3B heteromers (Fig. 1B). Addition of glutamate in the presence of glycine had no potentiating effect on current responses: the ratio of glycine-induced currents to glutamate/glycine-induced responses (I_Gly_/I_Glu/Gly_) was close to 1 for all splice variants tested (Fig. 1A). The I_Gly_/I_Glu/Gly _ratio of "conventional" NR1/NR2 combinations and separately expressed NR1 subunits was significantly lower and ranged between 0.01 for NR1-1a and 0.1 for NR1–3a (Fig. 1A).

To confirm that glycine-induced currents were indeed mediated by NR1/NR3 diheteromers, we extended the pharmacological characterization for the exemplary combinations of both NR1–3a and NR1–3b with either NR2B or NR3B. As described previously [7], desensitization of NR1/NR3 diheteromers occurred at glycine concentrations above 10 μM for both NR1 splice variants tested (data not shown, but see *Methods *section for values). This effect was not seen for NR1/NR2 diheteromers (data not shown). Furthermore, we tested two antagonists to confirm the formation of NR1/NR3 receptors. In these receptors, glycine binding to NR3 promotes channel opening (even if NR1 is unliganded [26]), while glycine binding to NR1 induces current decay [27]. Antagonists acting at either of the subunits therefore have a differential effect on the net response of the heteromer, a property we confirmed in the present study: CNQX, which binds to NR3 [27,28], blocked glycine-induced current responses of both NR1–3a/NR3B and NR1–3b/NR3B in agreement with previous studies [27], but displayed only weak effects on NR1–3a/NR2B and NR1–3b/NR2B receptors (Tab. 1). D-serine, a co-agonist of conventional NMDARs [29], also inhibited glycine-induced current responses of NR1–3a/NR3B diheteromers, as described previously for NR1–1a/NR3 [7]. Upon addition of 500 μM D-serine, glycine-induced current responses of NR1–3a/NR3B receptors were reduced by approximately 95% (I_Gly _= 592.2 ± 91.5 nA; I_Gly+D-Ser _= 30.7 ± 5.9 nA, n = 13).

**Table 1:** Antagonistic block of glycine-induced currents of exemplary diheteromers

**Agonist/antagonist** **(all conc. μM)**	**10 Gly/5 CNQX**	**10 Gly/100 KynA**	**150 Gly/100 KynA**
block of glycine-induced current response in % ± SEM			

NR1–3a/NR2B	10.3 ± 3.3	39.4 ± 1.6	47.5 ± 3.3

NR1–3b/NR2B	10.9 ± 5.8	29.4 ± 6.5	30.5 ± 5.1

block of glycine-induced current response in % ± SEM			

NR1–3a/NR3B	87.1 ± 1.7	84.6 ± 1.3	-244.9 ± 48.5

NR1–3b/NR3B	84.4 ± 1.5	82.1 ± 1.5	-263.9 ± 60.9

Overview of the block of glycine-induced current responses by CNQX and kynurenic acid (KynA) for exemplary NMDAR subunit combinations. Differences between NR1a- and NR1b- containing diheteromers are not statistically significant. n = 5 for each combination.

The NR1 glycine-site antagonist kynurenic acid, on the other hand, inhibited glycine-induced currents of NR1/NR3B receptors at low glycine concentrations (10 μM glycine, 100 μM kynurenic acid), but potentiated responses at higher agonist concentrations (150 μM glycine, 100 μM kynurenic acid) (Tab. 1). This pharmacological profile is unique for NR3-containing diheteromers, and in good agreement with previous studies employing the kynurenic acid derivatives 7-CKA and 5,7-DCKA [26,27]. We did not observe significant differences for NR1a and NR1b variants for either of the agonist/antagonist combinations tested here.

### The specific interaction with NR1 splice variants differs for NR2B and NR3B

**Figure 2:**
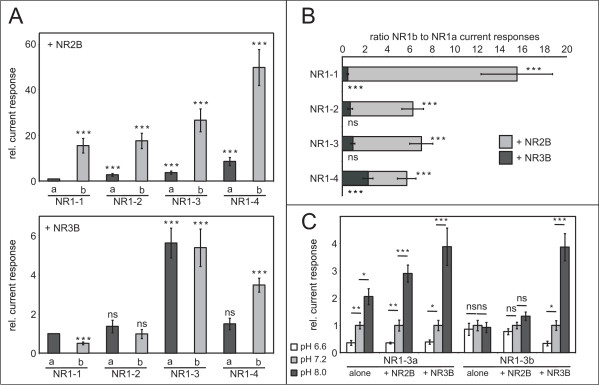
**Comparison of glutamate/glycine-induced steady-state current amplitudes for all NR1 isoforms with either NR2B or NR3B**. **A**. Relative current responses for each of the eight NR1 splice variants coexpressed with NR2B (upper panel) or NR3B (lower panel). Amplitudes were normalized to the responses mediated by NR1-1a/NR2B and NR1-1a/NR3B, respectively. All currents were recorded after the application of 300 μM glutamate and 10 μM glycine. Data shown here are mean values ± Gaussian error propagation, n = 6–10 from 2 independent experiments per combination. **B**. Current potentiation by the N-terminal NR1b splice variants over NR1a variants when coexpressed with either NR2B (light grey) or NR3B (dark grey). Responses of the NR1a variants were independently set to 1 and responses mediated by NR1b variants were normalized to the responses of the respective NR1a variants. Data are mean values ± Gaussian error propagation, n = 6–10 from 2 independent experiments per combination. ns = not significant. **C**. Relative glutamate/glycine-induced current responses of NR1–3a- and NR1–3b-containing NMDARs depending on the pH value. Current responses were normalized to the values measured at pH 7.2. Data are mean values ± Gaussian error propagation, n = 6. alone = expressed alone; ns = not significant.

The finding that NR3B is generally able to functionally interact with each NR1 splice variant prompted the question whether the current response of the NR1/NR3 heteromer is dependent on the C-terminal splicing of NR1 in the same manner as that of the conventional NR1/NR2 receptor. In "conventional" NMDARs, glutamate/glycine-mediated current amplitudes showed a dependence on the size of the C-terminal NR1 splice variant. Current levels increased along the sequence NR1-1 < NR1–2 < NR1–3 < NR1–4. Splice variants lacking exon 5 (NR1a variants) are tonically inhibited by protons at physiological pH values. Therefore, under the experimental conditions used, NR1a variants gave rise to generally lower current amplitudes, but showed the same dependence of amplitudes on the NR1 C terminus (Fig. 2A, upper diagram).

By contrast, amplitudes of glutamate/glycine-induced responses of NR1/NR3B receptors displayed a different pattern of dependency on the C-terminally spliced NR1 variants. To standardize conditions, and for better comparison, we here present currents measured after co-application of glutamate and glycine for all diheteromers. As NR1/NR3B receptors are fully activated by glycine, the additional presence of glutamate should not influence currents induced by application of glycine alone. Potentiation of current responses upon addition of glutamate occured only in oocyte batches with high expression levels of *Xen*NR2B (see below). These batches were excluded from quantitative comparison of current amplitudes; furthermore, all subunit combinations were measured in the same batches. Compared to the NR1-1a/NR3B receptor, statistically significant differences in current amplitudes were only observed for NR1-1b, NR1–3a, NR1–3b and NR1–4b. However, when compared to receptors featuring NR1-1a/NR2B, the NR1–3a/NR3B receptor was the only diheteromer displaying a similar increase in the relative current size (rel. increase in amplitudes compared to NR1-1a-containing heteromers: 3.78 ± 0.68 for NR1–3a/NR2B; 5.63 ± 0.76 for NR1–3a/NR3B; Fig. 2A).

Strikingly, the proton-dependent difference between NR1a and NR1b variants seen for current amplitudes of NR1/NR2 receptors could not be corroborated for all NR1/NR3B combinations (Fig. 2B). Significant differences were only seen for NR1-1/NR3B (with a factor of 0.5 between NR1a- and NR1b-containing receptors) and NR1–4/NR3B (factor: 2.3).

### NR3B-containing diheteromers are proton sensitive, regardless of the N-terminal NR1 isoform

The main difference between the N-terminal NR1 variants is their susceptibility to proton inhibition. As the influence of the NR1a and NR1b variants differed dramatically in complexes with NR2B and NR3B, we suspected that NR3B might interfere with proton inhibition. We chose the NR1–3a and NR1–3b subunits and compared current responses after the application of glutamate and glycine at three different pH values (pH 6.6, 7.2 and 8.0). As depicted in figure 2C, agonist-induced current responses of the NR1–3a subunit – expressed alone or together with either NR2B or NR3B – significantly increased with the pH value. By contrast, altered proton concentrations did not yield significantly different current responses if NR1–3b was expressed alone or together with NR2B. However, if NR1–3b and NR3B were coexpressed, agonist induced current responses increased with higher pH values, regardless of the presence of an NR1b isoform, suggesting that all NR3B-containing diheteromers are proton-sensitive.

### All NR3B-containing diheteromers are insensitive to block by Mg^2+ ^ions

**Figure 3:**
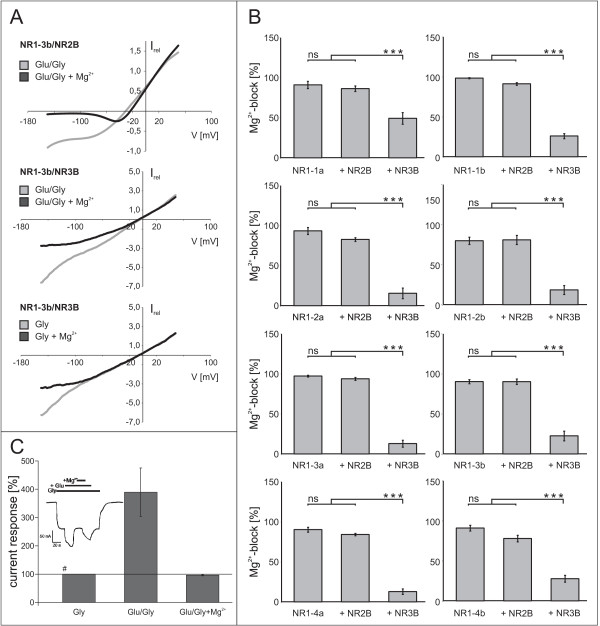
**Comparison of the Mg^2+ ^block of NR3B-containing diheteromers with each of the NR1 splice variants**. **A**. Current-voltage (IV) relationships for NR1–3b/NR2B (upper diagram, Glu/Gly-induced currents) and NR1–3b/NR3B (middle and lower diagrams for Glu/Gly- and Gly-induced currents, respectively) recorded between -150 mV and +50 mV. Recordings were performed in the presence of 300 μM glutamate (Glu) and/or 10 μM glycine (Gly) in the presence (dark grey traces) and absence (light grey traces) of 0.5 mM Mg^2+^. Traces represent averages from 4 experiments per combination (normalized to +20 mV). **B**. Comparison of the Mg^2+ ^block of agonist-evoked current responses at -70 mV for each NR1 splice variant expressed alone, with NR2B, or with NR3B. Mean values ± SEM, n = 9–18 from 2–5 independent experiments per combination; *p < 0.05; **p < 0.01; ***p < 0.005 (Student's t-test). **C**. Exemplary potentiation by glutamate of glycine-induced NR1–3b/NR3B receptor currents in oocytes with high expression levels of *Xen*NR2B. Note that only the portion of current induced by additional glutamate is blocked by Mg^2+^. Current responses are mean values ± SEM normalized to the glycine-induced response (set to 100% and marked with #), n = 6. The inset shows a representative current trace from this batch recorded during sequential applications of agonists and Mg^2+^.

NR1/NR3 diheteromers have been described to lower sensitivity to block by Mg^2+ ^ions compared to "conventional" NR1/NR2 NMDARs. We have asked whether this is true for heteromeric combinations with each of the eight functional splice variants of NR1. We therefore tested the Mg^2+ ^block of glycine- and glutamate/glycine-induced currents for all possible NR1/NR3B combinations. Shown in figure 3A (upper diagram) is the current-voltage relationship (IV) of the "conventional" NMDAR composed of NR1–3b/NR2B. Below -65 mV, agonist-induced currents were almost completely blocked in the presence of 0.5 mM Mg^2+^. For the NR1/NR3B diheteromer the block was less pronounced, and almost non-existent if only glycine was applied (compare Fig. 3A, middle and lower diagrams). The same held true for all possible combinations of NR3B with NR1 splice variants (IV's not shown). The Mg^2+ ^block at -70 mV is depicted in figure 3B for all NR1/NR3B heteromers in comparison to NR1 expressed alone and "conventional" NR1/NR2B receptors. Note that the extent of block for NR1/NR3B receptors varied between 12.9 ± 3.4% with NR1–4a and 49.4 ± 7.3% with NR1-1a. However, this cannot be attributed to a specific influence of NR1 variants on NR3B-containing NMDARs, but rather constitutes a peculiarity of the oocyte expression system. As has been shown recently [30], homomerically expressed exogenous NR1 assembles with the endogenous *Xen*NR2B subunit in oocytes to form functional NMDARs with properties similar to receptors from exogenous, mammalian NR1/NR2 subunits.

In the case of coexpressed NR1 and NR3B in oocytes, the presence of an additional, endogenous, glutamate-binding, Mg^2+^-sensitive subunit such as *Xen*NR2B has the potential to distort results. As shown in Fig. 3C for the combination NR1–3b/NR3B, in oocyte batches with high expression of *Xen*NR2B, glycine-induced currents could be potentiated by the addition of glutamate. This additional current could in turn be completely and reversibly blocked by Mg^2+^, whereas the glycine-evoked fraction of the response remained insensitive to Mg^2+ ^ions (Fig. 3C). Similar effects were seen with ifenprodil and APV (data not shown). For the comparative study of Mg^2+ ^block of NR1/NR3B heteromers, it was not always possible to choose oocyte batches with expression levels of *Xen*NR2B below detection threshold. Therefore, Mg^2+ ^blocks are even lower than depicted in Fig. 3C, and a quantitative comparison between subunits cannot be performed. However, with glycine as the sole agonist, the Mg^2+^-induced block was considerably lower. The fractions of currents blocked by Mg^2+ ^in receptor complexes assembled from NR3B and one of the eight NR1 splice variants were as follows (in %): NR1-1a: 12.9 ± 5.3; NR1-1b: 16.7 ± 5.0; NR1–2a: 12.6 ± 4.1; NR1–2b: 5.3 ± 5.3; NR1–3a: 3.1 ± 1.6; NR1–3b: 11.6 ± 4.1; NR1–4a: 10.5 ± 3.0; NR1–4b: 9.8 ± 3.3, n = 11–18.

## Discussion

NMDA receptors *in vivo *are at the molecular core of complex cognitive functions, most prominently via their involvement in LTP. Consequently, dysfunctional NMDARs are implicated in various pathological conditions. Most of the conventional NMDARs' functional characteristics are governed by their unique coincidence detection mechanism: the requirement of two agonists plus a simultaneous voltage-dependent release of their Mg^2+ ^block for receptor activation. As NR3 alters each of these critical traits by forming excitatory glycine receptors with NR1, and as NR3 is potentially diverting NR1 from classical NR1-NR2 NMDA receptors, the function of classical NMDARs will invariably be impacted.

Consequently, NR3B has been discussed to influence cell death in the spinal cord [10] or specifically protect somatic motoneurons [8] – cells that selectively succumb to glutamatergic excitotoxicity in neurodegenerative disorders like Amyotrophic Lateral Sclerosis (ALS). Along this line, slightly impaired motor-learning has been reported in NR3B-deficient mice [31].

The question whether NR1/NR3 receptors indeed have a functional impact *in vivo *hinges on whether these diheteromers actually occur *in vivo*, a question that is not yet satisfactorily answered. We contribute to the elucidation of this matter by showing that NR3B is able to recruit every single functional isoform of the NR1 subunit into a diheteromer – rendering the occurrence of such receptors in the vertebrate CNS very likely.

### NR3B can functionally interact with all eight NR1 splice variants to form Mg^2+^-insensitive diheteromeric receptors

The NR1 isoforms are in part responsible for the heterogeneity of NMDAR properties *in vivo*: N-terminal splicing determines proton sensitivity [13], while C-terminal splice variants influence synaptic plasticity by modulating activity-dependent trafficking of isoforms [32]. However, the data presented here suggest this to be true only for the "conventional" NR1/NR2 diheteromers. While the assembly of NR1 and NR3 is well established for a limited set of subunit combinations [7,24,33], we have for the first time compared the interaction of NR3B with each of the eight functional NR1 splice variants. In all cases functional receptors assembled, which were fully activated by glycine alone, but were insensitive to the addition of glutamate. Between splice variants, small differences were seen in the I_Gly_/I_Glu/Gly _ratios. However, these cannot necessarily be attributed to the influence of the different NR1 isoforms. Rather, small variations may result from the interaction of the endogenously expressed *Xen*NR2B subunit [30] with exogenous NR1. The amount of *Xen*NR2B varies for different batches of oocytes, which likely explains the differences in I_Gly_/I_Glu/Gly _ratios.

The influence of endogenous *Xen*NR2B also effects the extent of the Mg^2+^-block of NR1/NR3B diheteromers. Glycine-induced current responses of NR1/NR3B receptors were hardly blocked by Mg^2+^, which is in accordance with previous studies on NR1-1a/NR3B receptors [7]. We have shown here that there is no dependence of Mg^2+ ^sensitivity on the NR1 isoform. Rather, NR3B lowers Mg^2+ ^sensitivity of the diheteromeric receptors regardless of the NR1 variant. This finding is unsurprising, as the Mg^2+ ^block depends on residues within the pore region of NMDAR subunits that do not differ for the eight NR1 splice variants.

### The NR1 isoform-specific interaction with NR2B cannot be corroborated for NR3B

NR3 requires NR1 for export from the endoplasmatic reticulum (ER) [34]. ER export, in turn, is dependent on the C-terminus of the NR1 subunit, as has been shown for conventional NR1/NR2 receptors [19,20]. Under the standardized conditions of the heterologous expression system, current amplitudes are determined by the number of NMDARs in the cell membrane, and therefore the export levels from the ER. For the conventional NR1/NR2B heteromers, current levels increased as the size of the NR1 C-terminus decreased, with the proton-inhibited NR1a variants mediating generally smaller current amplitudes at pH 7.2. The presence of NR3B instead of NR2B in the diheteromer altered this dependency dramatically: While some NR1 isoforms mediated current responses that were statistically significantly larger compared to NR1-1a, they range on a tenfold lower scale compared to current responses at NR1/NR2 receptors. It therefore remains questionable whether the observed variability is of physiological relevance. Rather, NR3B appears to interact comparably with all eight NR1 isoforms in terms of trafficking efficiency. Interestingly, the largest agonist-induced current responses were mediated by diheteromers featuring the NR1–3 splice variants – the isoforms with the lowest transcript levels in the developing and adult rodent brain [22,23].

### All NR3B-containing diheteromers are proton-sensitive

We have shown that the presence of NR3B in a diheteromer erases any differences in proton sensitivity between NR1a and NR1b variants. In the conventional NR1/NR2B receptor, the additional protein loop encoded by exon 5 of the NR1b subunits shields the proton-sensitive regions [13,14,35,36], rendering the receptor insensitive to proton inhibition. Recent studies suggest the alignment of the pore-lining regions of NR1 and NR3A to be structurally distinct from that of NR1 and NR2 [37]. It is entirely conceivable that even a slightly different assembly of NR1/NR3 diheteromers compared to NR1/NR2 disturbs the specific alignment of the protein loop encoded by exon 5 of NR1b and renders it ineffective as a proton shield. Physiologically, this finding supports the general notion of NR3 as a dominant-negative modulator of NMDAR function [8,34,38]. Our data extend this notion to diheteromers composed of NR1 and NR3B. If NR1/NR3 receptors exist *in vivo*, they will invariably be inhibited by protons at physiological pH values, irrespective of the NR1 isoform. This is especially interesting in the light of mRNA expression patterns of the subunits concerned: in the adult rodent brain, NR1b variants have been indicated to be expressed more abundantly in cerebral subdivisions and structures concerned with motor control and have been suggested to play a role in motor coordination [23]. Interestingly, a similar role has been proposed for the NR3B subunit, as its expression in the adult mouse is limited to somatic motor neurons of cranial nerve nuclei and the anterior horn of the spinal cord [8]. Considering the specific excitotoxic damage to motor areas and motoneurons in certain pathological conditions, it is tempting to speculate whether a tight regulation of NMDAR activity is necessary in these areas and cells. Such a regulation could be achieved by tonically inhibiting NMDARs at physiological pH values.

## Conclusion

A key feature of NMDARs is their heterogeneity *in vivo*. We provide evidence that NR3B assembles with each NR1 splice variant to form functional receptors that share the unique properties of excitatory glycine receptors described for NR1-1a/NR3 diheteromers. However, the specific functional properties determined by the NR1 isoform in conventional NR1/NR2 receptors did not hold up for assembly with NR3. By contrast, neither receptor trafficking (mediated by the NR1 C terminus) nor insensitivity to proton inhibition (mediated by an N-terminal structural motif) are dependent on the NR1 isoform in NR1/NR3B complexes. Rather, NR3B appears to override NR1 splice variant-dependent effects. These results substantiate the view of NR3B as a downregulator of agonist-induced NMDAR current responses by establishing this subunit in NR1/NR3B receptors as an "equalizer" of NMDAR action through NR3B-mediated block of the proton insensitivity normally conferred by NR1b splice variants. This suggests that the precisely orchestrated NR1 variant-dependent receptor properties are tempered in cells with high expression of NR3B in favor of a general dominant-negative influence of this subunit. Future studies will have to take into consideration how this behavior affects triheteromeric (NR1/NR2/NR3) NMDARs.

## Abbreviations

5,7-DCKA: 5,7-dichlorokynurenic acid; 7-CKA: 7-chlorokynurenic acid; ALS: amyotrophic lateral sclerosis; CNQX: 6-cyano-2,3-dihydroxy-7-nitro-quinoxaline; CNS: central nervous system; D-Ser: D-serine; ER: endoplasmatic reticulum; glu: glutamate; gly: glycine; iGluR: ionotropic glutamate receptor; KynA: kynurenic acid; LTD: long-term depression; LTP: long-term potentiation; NMDA: N-methyl-D-aspartate.

## Authors' contributions

NAC and AO carried out the electrophysiological measurements and performed the statistical analyses; NAC drafted the manuscript. MH participated in the design and supervision of the study and writing of the manuscript. All authors read and approved the final manuscript.
